# Long-term hospital-based secondary prevention of coronary artery disease: a randomized controlled trial

**DOI:** 10.1186/s12872-021-02426-3

**Published:** 2021-12-16

**Authors:** Anete Kaldal, Serena Tonstad, Jarle Jortveit

**Affiliations:** 1 Department of Research, Sørlandet Hospital HF, Lundsiden, Box 416, 4604 Kristiansand S, Norway; 2 Department of Endocrinology, Obesity and Preventive Medicine, Section of Preventive Cardiology, Oslo University Hospital, Oslo, Norway; 3 Department of Cardiology, Sørlandet Hospital Arendal, Arendal, Norway

**Keywords:** Myocardial infarction, Cardiovascular diseases, Secondary prevention

## Abstract

**Background and aims:**

Despite established guidelines on secondary prevention of cardiovascular disease, practical implementation of treatment targets is deficient even in high-income countries. This study compared long-term hospital-based treatment with follow-up at primary health care regarding new cardiovascular events and achievement of treatment targets.

**Methods:**

This randomized controlled trial at Sørlandet Hospital, Norway 2007–2021 included patients hospitalized due to myocardial infarction (n = 760) or after scheduled percutaneous coronary intervention (PCI) (n = 677) or coronary artery bypass grafting (n = 103). Patients were randomized to hospital-based secondary preventive care with consultations 2 weeks, 3 months, 6 months and 1 year after the index event and annually for up to 5 years, or follow-up at primary health care. Final data was collected after 10 years and hazard ratios were calculated using Cox regression analyses.

**Results:**

Composite endpoint-free survival due to a lower rate of PCI improved in patients with hospital-based follow-up (n = 788) compared to patients followed-up at primary health care (n = 752) (HR 0.80, 95% CI 0.66–0.96; *p* = 0.02) but all-cause mortality was not reduced (HR 0.96, 95% CI 0.59–1.56; *p* = 0.86). At 1 year, LDL-cholesterol (2.1 [SD 0.7] versus 2.3 [SD 0.8] mmol/l; *p* < 0.001) and systolic blood pressure (132 [SD 16] versus 142 [SD 20] mm/Hg; *p* < 0.001) were lower in the hospital-based group, and the differences remained significant during the first 5 years. Other secondary preventive measures (smoking cessation, physical activity, body weight, glucose control, drug adherence) did not differ.

**Conclusions:**

Long-term hospital-based secondary preventive follow-up improved composite endpoint-free survival, but not mortality. Substantial risk factors remained unaddressed. The beneficial effects on blood pressure and LDL-cholesterol disappeared after annual consultations ceased.

*Trial registration*: The study is registered in ClinicalTrials.gov (NCT00679237) May 16, 2008.

**Supplementary Information:**

The online version contains supplementary material available at 10.1186/s12872-021-02426-3.

## Introduction

The significance of the modifiable risk factors in development of cardiovascular disease (CVD) is well documented [1, 2]. Secondary preventive measures focusing on adequate medical treatment and lifestyle modification could prevent recurrent cardiovascular events [3]. The European Society of Cardiology (ESC) and American Heart Association (AHA)/American College of Cardiology Foundation (ACCF) have issued detailed guidelines on secondary prevention of CVD [4, 5]. However, large studies such as EUROpean Action on Secondary and Primary prevention through Intervention to Reduce Events (EUROASPIRE) I, II, III and IV [6, 7], as well as the prospeCtive observational LongitudinAl RegIstry oF patients with stable coronary arterY disease (CLARIFY) [8] and the REduction of Atherothrombosis for Continued Health (REACH) [9] demonstrate a remaining gap between the guidelines and the achievement of recommended goals even in high-income countries.

In Norway, approximately 12 000 men and women are diagnosed with myocardial infarction (MI) [10] and approximately 14 000 procedures of percutaneous coronary interventions (PCI) or coronary artery bypass grafting (CABG) are performed annually [11]. Approximately 30% of MIs occurred in patients with prior MI [10]. A recent nationwide study based on the Norwegian Myocardial Infarction Register showed that only 1% of MI patients with established coronary artery disease (CAD) reached all secondary prevention treatment targets, and only half of these were attained on average [12]. Similarly The NORwegian CORonary Prevention Study (NOR-COR) found that on average three of six major risk factors were not attained, and patients with more than one coronary event had poorest achievement of treatment targets [13]. Although the health care system in Norway is well developed, there is an inequality on national basis regarding the follow-up of CAD patients, including differences in medical treatment as well as participation in cardiac rehabilitation programs [13–15]. Hospital-based follow-up is missing at many hospitals or is based on relatively short-term rehabilitation programs or “heart schools”. The questions whether hospital-based long-term follow-up contributes to better modifiable risk factor control and how it affects morbidity and mortality are still unclear.

The aim of the present study was to investigate the effects of long-term hospital-based secondary prevention follow-up program in patients with CAD.

## Methods

### Study design and study population

The study was conducted as an open, randomized, controlled trial in accordance with Declaration of Helsinki at Sørlandet Hospital Arendal, Norway in the period 2007–2021. Consecutive patients admitted to the hospital with a diagnosis of MI or after scheduled PCI/CABG aged 18–80 years were randomized to the intervention arm of the study (hospital-based follow-up) or to follow-up within primary health care. A simple randomization was performed by random number generator prior to study start, and the study nurses were responsible for screening, inclusion and obtaining informed consent. Exclusion criteria were lack of ability to cooperate, known alcohol- or drug-abuse, use of narcotics, pregnancy or breast-feeding, serious comorbidity with a life expectancy less than 2 years, or participation in other secondary prevention studies. Patients not randomized to the intervention arm were, after 1 year, formally asked to participate as controls. The exclusion criteria were the same and written consent was required for both groups. This design was chosen to avoid confounding by the patients knowing that they were in a control group in a study. Patients participating in this study were enrolled between September 2007 and January 2017.

### Intervention (hospital-based follow-up)

**Fig. 1:**
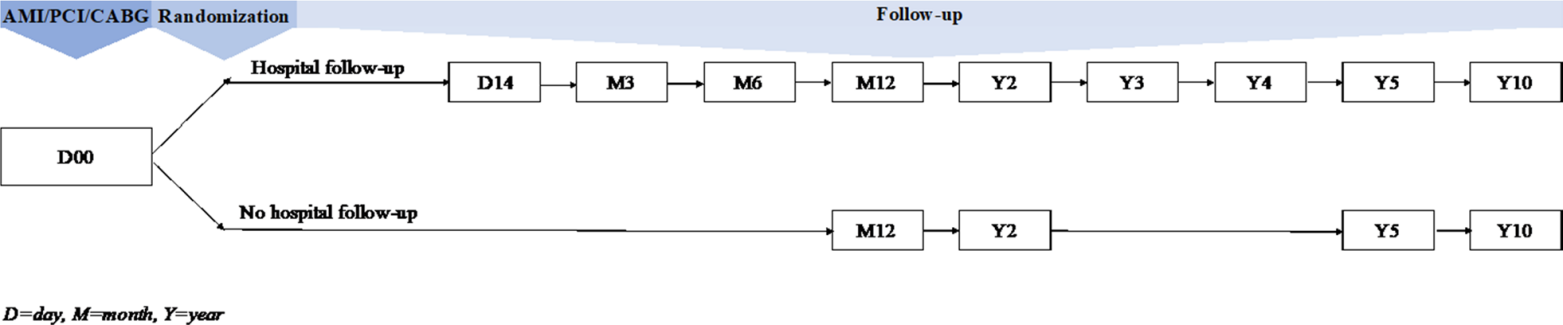
Study flow chart

Regular outpatient consultations were offered for patients in the hospital-based follow-up group. Specially trained nurses, supervised by cardiologists, followed up patients starting from the first consultation during the hospital admission for the index event (MI or PCI/CABG), thereafter at 2 weeks, 3 months, 1 year and annualy for up to 5 years. Final data were collected after 10 years (Fig. 1). Total risk reduction was in focus for entire follow-up period, and treatment goals were explained to the patients to facilitate concordance and compliance. The attainment of goals was evaluated at each consultation, and following measures were assessed: blood pressure, weight, height, waist circumference, LDL-cholesterol and HbA_1c_. Smoking status (daily/occasionally/previously (≥ 1 month)/no smoking history) and use of medication were reported by the patient. In addition, date and treatment of MI or PCI/CABG were registered at study start. At each consecutive consultation data about death, hospital admissions, stroke, recurrent MI or new PCI/CABG were recorded.

#### Intervention measures

- Smoking cessation: Nicotine replacement therapy (NRT) was offered during hospital admission, and continuation of NRT or a 12-weeks course of varenicline after discharge was advised.
- Blood pressure: In addition to the promotion of weight reduction, increased physical activity and dietary measures, pharmacological antihypertensive therapy was initiated and/or adjusted. The choice of medication was based on an individual clinical evaluation of each patient.
- All the participants were prescribed statins unless contraindicated, and other lipid lowering agents (primarily ezetimibe) were added to treatment if statins alone did not provide recommended result.
- Patients with a diagnosis of diabetes mellitus were identified and antidiabetic therapy was initiated and/or adjusted after clinical evaluation.
- All patients were referred to organized training program once a week for 3 months supervised by physiotherapists. Physical activity of moderate intensity ≥ 150 min weekly or of vigorous intensity ≥ 75 min weekly was advised to all participants. Continuation of physical activity on individual basis after ended training program was encouraged.
- SmartDiet^tm^ [16] scoring was used to assess dietary habits. Individual nutritional guidance was provided based on the responses and aimed at improvement of lipid profile.
- Acetylsalicylic acid (ASA) and adenosine diphosphate (ADP) receptor inhibitors were prescribed accordingly to clinical guidelines.
- Additional medication (e.g. β-blockers and renin–angiotensin–aldosterone system inhibitors) was prescribed as appropriate for the actual treatment setting.

All patients who are discharged from the hospital after acute myocardial infarction or PCI/CABG—regardless of participation in the study or not—are advised concisely regarding secondary preventive measures as a routine. All the patients are advised as well regular follow-up at primary health care. For patients who participated in the study and were randomized to the control group, follow-up with respect to the secondary preventive measures was in the charge of family physician, and only data collection was carried out through short outpatient consultations without intervening in the treatment regimes or giving further life style recommendations. The first consultation was 12 months after discharge, thereafter at 2 years, 5 years and final data collection at 10 years (Fig. 1). Medical records from the index event were used as baseline information of the participant. For patients who were randomized to hospital-based follow-up regular visits at family physician were important to maintain continuity of information flow between the secondary health care, patient and the family physician, as well as to ensure that the patients are followed up regarding other conditions and diseases, while outpatient consultations had a major role in the follow-up of secondary preventive measures as guidance of patient with regard to lifestyle amendments and adjustment of medical treatment.

#### Treatment targets of secondary prevention

The secondary preventive treatment targets adhered to the latest ESC guidelines available [4, 17–22].

- No smoking
- Blood pressure < 140/90 mmHg
- LDL-cholesterol < 1.8 mmol/l (< 2.5 mmol/l until 2017, < 1.4 mmol/l from 2020)
- HbA_1c_ < 53 mmol/mol (7%)
- BMI < 25 kg/m^2^
- Daily use of statins
- Daily use of acetylsalicylic acid
- Physical activity of moderate intensity ≥ 150 min weekly or of vigorous intensity ≥ 75 min weekly

### Outcomes

The primary endpoints were all-cause mortality and a composite of all-cause mortality, PCI, CABG, non-fatal stroke or non-fatal MI (first event) during the follow-up. The secondary endpoints were proportion of participants who attained the secondary preventive treatment targets.

### Statistical analysis

Continuous variables are reported as means ± SD (standard deviations) and differences between groups were analysed using independent samples t-tests. Categorical variables are presented as numbers and percentages, and differences between groups were analysed by the chi-squared test. Adjustments for multiple comparisons were not applied. Kaplan–Meier curves for crude and composite endpoint-free survival after hospital admission for the first MI or PCI/CABG in the study period were estimated. Cox regression analyses were used to calculate age-adjusted hazard ratios (HRs) with 95% confidence intervals (CIs) for endpoints. The sample size was calculated based on the Vestfold Heartcare Study.^23^ A p-value of < 0.05 was regarded as statistically significant. The statistical analyses were performed by using STATA, version 16.1 (StataCorp, 4905 Lakeway Dr, College Station, TX 77,845, USA).

## Results

**Fig. 2:**
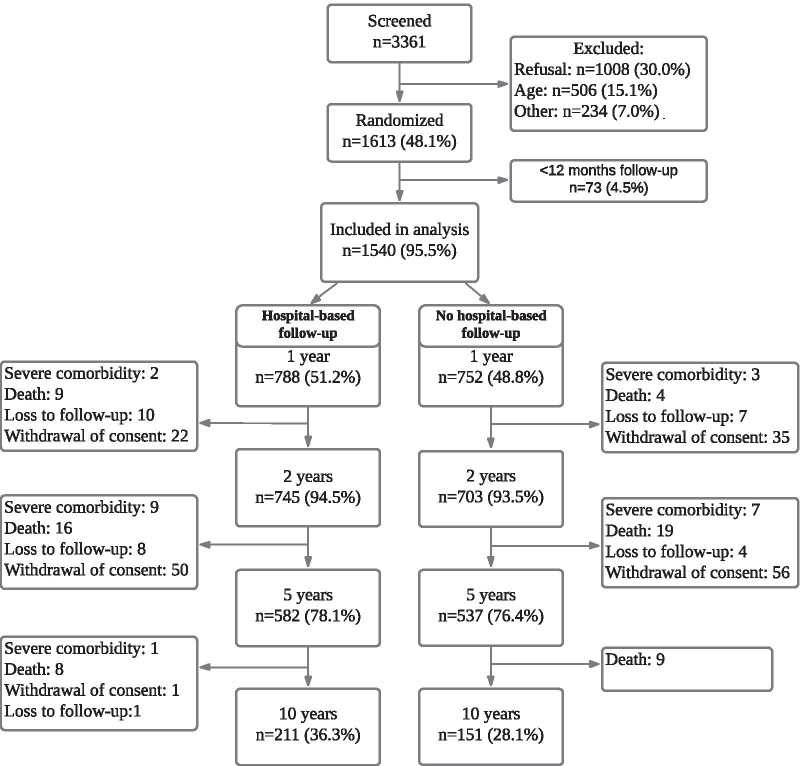
Number of patients at screening, randomization, inclusion in data analysis and participating in study at each follow-up consultation

A total of 3361 patients were screened during the inclusion period 2007–2017, 1613 (48.1%) patients were included in the study, 1008 (30.0%) of patients refused participation, 506 (15.1%) were excluded due to age and 234 (7.0%) could not participate due to other reasons such as lack of ability to cooperate, drug abuse, participation in other studies or short life expectancy (Fig. 2). Due to the inclusion of patients in the control (no hospital-based follow-up) group 12 months after the index event, 73 patients in the intervention (hospital-based follow-up) group with < 12 months follow-up, were excluded from further analysis. A total of 1540 patients (788 (51.2%) patients in the intervention group and 752 (48.8%) patients in the control group) were included in the data analysis. During the study period from September 2007 until April 2021, 8082 outpatient consultations were conducted.

### Clinical characteristics

Baseline characteristics are described in Table 1. A total of 205 (13%) patients had a history of prior MI, 209 (14%) prior PCI, 91 (6%) prior CABG and 73 (5%) patients had experienced a stroke. 178 (23%) and 159 (21%) of the patients were women in the group with and without hospital-based follow-up, respectively. Half of the patients (n = 394 (50%)) in the hospital-based follow-up group and 366 (49%) of the patients in the primary health care group were hospitalized due to acute MI. A total of 334 (42%) patients and 343 (46%) patients underwent PCI (without acute MI), while 60 (8%) and 43 (6%) patients were treated with CABG, respectively. Mean age was lower (62 [SD 10] versus 64 [SD 9] years) in the hospital-based follow-up group, otherwise we found no differences in baseline characteristics.

**Table 1:** Baseline clinical characteristics at hospitalization for index event in patients with and without hospital-based secondary preventive follow-up program after acute myocardial infarction (MI), percutaneous coronary intervention (PCI) or coronary artery bypass grafting (CABG)

	Hospital-based follow-up	No hospital-based follow-up	*p*
	n = 788	n = 752	
	n	n	
Mean age (years) (SD)	62 (10)	64 (9)	< 0.001
Male	610 (77%)	593 (79%)	0.50
Higher education*	205 (28%)	201 (29%)	0.58
Working**	304 (39%)	282 (38%)	0.33
Married/cohabiting	624 (79%)	600 (80%)	0.79
Mean body mass index (kg/m^2^) (SD)	28 (5)	28 (4)	0.15
Smoking	224 (28%)	203 (27%)	0.57
Lipid lowering therapy	346 (44%)	333 (44%)	0.80
Antihypertensive therapy	367 (47%)	355 (47%)	0.96
Diabetes	120 (15%)	110 (15%)	0.72
Previous coronary heart disease:			
Myocardial infarction	106 (13%)	99 (13%)	0.82
Percutaneous coronary intervention	106 (13%)	103 (14%)	0.94
Coronary artery bypass grafting	45 (6%)	46 (6%)	0.75
Previous stroke	36 (5%)	37 (5%)	0.81
Mean LDL-cholesterol (mmol/L) (SD)	3.0 (1.1)	2.9 (1.1)	0.29
Mean systolic blood pressure (mmHg) (SD)	145 (24)	147 (24)	0.10
Mean diastolic blood pressure (mmHg) (SD)	86 (14)	87 (14)	0.50
Mean left ventricular ejection fraction (%)(SD)	52 (9)	51 (10)	0.48
Qualifying diagnosis:			
Myocardial infarction	394 (50%)	366 (49%)	0.61
Percutaneous coronary intervention	334 (42%)	343 (46%)	0.22
Coronary artery bypass grafting	60 (8%)	43 (6%)	0.15

*Higher education: college and/or university education

**Working: engaged in paid employment

### Outcomes

#### Primary outcomes

**Fig. 3:**
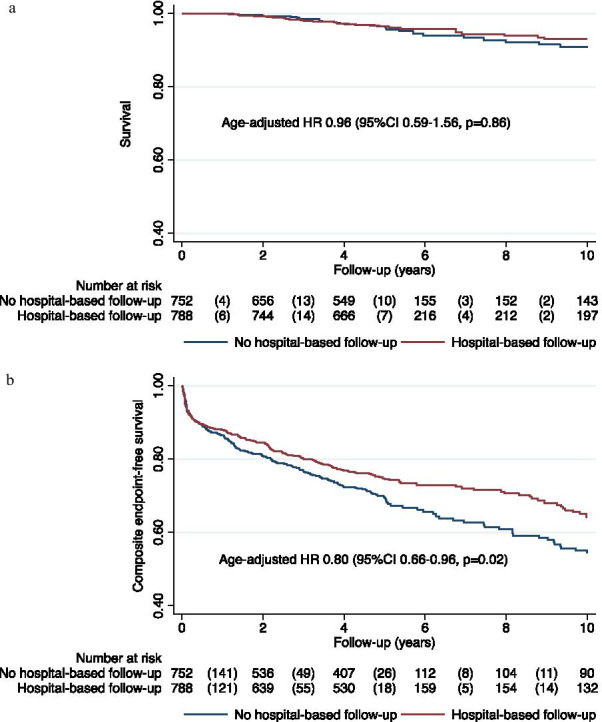
Survial (**a**) and composite endpoint-free survival (**b**) and in patients with and without hospital secondary preventive follow-up program after myocardial infarction (MI), percutaneous coronary intervention (PCI) or coronary artery bypass grafting (CABG)

After a median follow-up time of 1837 days (25th percentile 1552 days, 75th percentile 2012 days), 48 (3%) patients had died. We found no significant differences between the groups regarding all-cause mortality, and the survival calculated by Cox-regression resulted in age-adjusted HR 0.96, 95% CI 0.59–1.56; *p* = 0.86 (Table 2 and Fig. 3a). The median time to death was 1255 days and 1410 days in the group with and without hospital-based follow up, respectively. The proportion of women (14 (4.2%)) and men (51 (4.2%)) who died during the follow-up was similar (*p* = 0.95). The median age for those who died was 77.9 years (25th percentile 69.2, 75th percentile 77.6 years), and the mean age at death did not differ significantly between the follow-up groups (*p* = 0.65) and genders (*p* = 0.65).

**Table 2:** All-cause mortality, composite endpoint and total number of cardiovascular events in patients with and without hospital-based secondary preventive follow-up program after acute myocardial infarction (MI), percutaneous coronary intervention (PCI) or coronary artery bypass grafting (CABG)

	Hospital-based follow-up	No hospital-based follow-up	*p*
	n = 788	n = 752	
	n	n	
All-cause mortality	33 (4%)	32 (4%)	0.86
Composite endpoint*	214 (27%)	235 (31%)	0.02
Myocardial infarction	38 (5%)	39 (5%)	0.56
Percutaneous coronary intervention	144 (18%)	179 (24%)	0.002
Coronary artery bypass grafting	11 (1%)	12 (2%)	0.71
Stroke	33 (4%)	30 (4%)	0.95

Mean follow-up time was 5.9 [SD 2.8] and 5.3 [SD 2.8] years in the group with and without hospital-based follow-up, respectively (*p* < 0.001)

*Composite endpoint consists of all-cause death, non-fatal myocardial infarction, new PCI/CABG or non-fatal stroke, the first event determining the end of follow-up in the composite endpoint-free survival analysis

Fewer patients with hospital-based follow-up underwent new PCI (144 (18%) vs. 179 (24%), HR 0.71, 95% CI 0.57–0.88; *p* = 0.002) and composite endpoint-free survival was significantly higher (age-adjusted HR 0.80, 95% CI 0.66–0.96; *p* = 0.02) in patients with hospital-based follow-up (Fig. 3b). Median time until new PCI was 147 days (25th percentile 27 days, 75th percentile 790 days) days and 263 days (25th percentile 42 days, 75th percentile 769 days) days in the groups with and without hospital-based follow-up, respectively. We found no significant differences between the groups regarding recurrent MI (HR 0.87, 95% CI 0.56–1.37; *p* = 0.56), CABG (HR 0.85, 95% CI 0.38–1.95; *p* = 0.71) or stroke (HR 0.98, 95% CI 0.60–1.62; *p* = 0.95).

#### Secondary outcomes

**Fig. 4:**
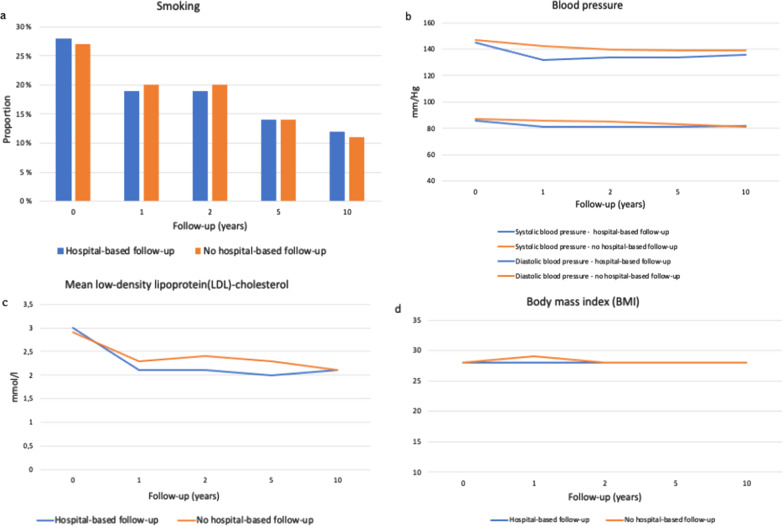
Proportion of smokers (**a**), blood pressure (**b**), LDL-cholesterol levels (**c**) and mean body mass index (BMI) (**d**) during study follow-up

Secondary endpoints are presented in Additional file 1: Table S1. We found no significant differences between the study groups regarding proportion of patients smoking throughout the observation period (Additional file 1: Table S1, Fig. 4a). A total of 224 (28%) and 203 (27%) patients with and without the hospital-based follow-up group reported smoking at the baseline, respectively. 88 (39%) smokers in the hospital-based follow-up group quitted within first two weeks after the index event. 24 (27%) of them had a relapse at 1 year follow-up. A total of 88 (39%) and 62 (31%) smokers reported abstinence from smoking after 1 year in the group with and without hospital-based follow-up, respectively, and of those 29 (33%) and 20 (32%) smoked again at 5 year consultation. Of the total, 21 patients reported use of NRT or varenicline in the hospital-based follow-up while 24 patients reported use of these in the primary health care group at 1 year follow-up. After 5-years 78 (35%) of the smokers in the intervention group and 60 (30%) smokers in the control group reported smoking cessation. Education level did not differ significantly among those who managed to quit within 5 years. Significantly fewer patients with higher (college/university) education reported smoking compared to those with basic (primary- or highschool) education, but there were no gender differences.

Mean blood pressure levels were significantly lower in the intervention group compared to the control group in the first 5 years of follow-up (Additional file 1: Table S1, Fig. 4b). However, there were no significant differences after 10 years. Except for the first year follow-up where significantly fewer patients in the control group received calcium channel blockers (19% vs. 24%, *p* = 0.02), there was no significant difference in proportion of patients receiving these, β-blockers or renin–angiotensin–aldosterone system inhibitors up to 5 years.

LDL-cholesterol levels were significantly lower in the hospital-based follow-up group until annual consultations ceased (Additional file 1: Table S1, Fig. 4c). Significantly higher proportion of patients in the hospital-based follow-up group received statins during the first 2 years (Additional file 1: Table S1). After 1 year 153 (20%) of patients in the hospital follow-up group and 42 (7%) patients who received usual care reported use of ezetimibe (*p* < 0.001), and there was even greater difference after 5 years (234 (41%) vs. 71 (15%), *p* < 0.001) and 10 years (95 (45%) vs. 28 (19%), *p* < 0.001).

The mean BMI was nearly constant in both groups and did not differ significantly between groups throughout the study (Additional file 1: Table S1, Fig. 4d). Proportion of patients exercising minimum 150 min/week was significantly higher in the hospital-based follow-up group during the first 2 years compared to the group without hospital-based follow-up (Additional file 1: Table S1). SmartDiet^tm^ score was significantly higher in the hospital-based follow-up group after 1 year (32 [SD 5] vs. 29 [SD 4], *p* < 0.001), and the difference remained significant up to the final consultation.

A total of 230 (15%) patients had diabetes mellitus (Table 1), while 48 (3%) patients without history of diabetes mellitus qualified for this diagnosis at the hospitalization for index event, and 179 (12%) developed diabetes during the follow-up. The proportion of diabetes patients reaching HbA_1c_ target was low and did not differ significantly between the groups (Additional file 1: Table S1).

Proportion of patients using acetylsalicylic acid and statins was significantly higher in the hospital-based follow-up group after 2 years, but the proportion declined in both groups over the study period (Additional file 1: Table S1).

## Discussion

This randomized controlled interventional trial at Sørlandet Hospital, Arendal, Norway 2007–2021 showed significant improvement of composite endpoint-free survival in patients with hospital-based follow-up after MI/PCI/CABG due to lower rate of new PCI compared to patients with no hospital-based follow-up.

While earlier meta-analyses on cardiac rehabilitation indicate reduction of all-cause and cardiovascular mortality [24, 25], a recent meta-analysis by van Halewijn et al. found no reduction in all-cause mortality [26]. Low general all-cause mortality might explain the similiar finding of equal survival in both groups in our study too.

Significantly higher composite endpoint-free survival in the hospital-based follow-up group was mainly due to lower rate of new PCI procedures. Reduced incidence of MI, reduced CVD related mortality [27, 28] and increased availability of PCI might replace MI and deaths with revascularization as the main outcome measurement.

The proportion of smokers decreased similarly in both groups over the study period. There are several aspects which may contribute to low effect of the intervention. Experience of cardiovascular event itself is a turning point for many smokers to change their attitude on smoking cessation [29, 30], and would give the same impact in both groups. Immediate cessation after acute coronary event seems to be the most significant predictor for successful quitters [31]. Thus, more emphasis on individual approach in offering medications and assistance available at the hospital while the patient is admitted for the index event [29] could possibly increase quitting rates. Furthermore, patients who manage to quit immediately after acute coronary event seem to have limited benefit of follow-up to avoid relapse [31]. Our results support several other studies, showing limited effect of outpatient secondary prevention programs on smoking cessation in patients who did not quit smoking immediately [32, 33].

Blood pressure and LDL-cholesterol levels were significantly lower in the hospital-based follow-up group, however, the effect disappeared after the cessation of annual consultations. We observed higher proportion of patients reaching the LDL-cholesterol treatment goal in both groups than previously described in Norway [34]. A number of studies corroborate our results, presenting significant improvement in lipid profile and blood pressure control during intervention [26, 33]. However, there is less confidence regarding the effect of the follow-up after the end of study period, as the control of risk factors tend to decline over time [33, 35, 36]. Our study underlines the importance of continued regularity of consultations to maintain the treatment results. A novel treatment options with proprotein convertase subtilisin/kexin 9 (PCSK9) inhibitors have demonstrated significantly lower LDL-cholesterol level in comparison to standard therapy alone with statins and/or ezetimibe [37, 38], reduction of cardiovascular events [37], substantial improvement in adherence to treatment regimen [39], as well as positive impact on the quality of life [40]. In our study only few participants received therapy with PCSK9 inhibitors, as the treatment is restricted to patients who do not reach optimal LDL-cholesterol level with maximally tolerated dose of statins and/or other lipid-lowering medicaments and those with inherited hypercholesteroleamia. Thus, the treatment with PCSK9 inhibitors didn´t affect the results of our study, but leaves a space for further contribution in the secondary prevention of CVD if the availability of these medicaments would increase.

The proportion of patients with diabetes who reached optimal glucose control in EUROASPIRE IV was 54% and 49% for men and women, respectively, after median follow-up of 1.4 years [7], thus indicating rather poor glycemic control in patients with diabetes in our study. Likewise, the Norwegian NOR-COR study found that only 41% of patients achieved glucose treatment goals after a median follow-up of 1.7 years [13]. The initial decline in the proportion of patients reaching HbA_1c_ treatment target in our study might be partially explained with the fact that at least 4% of patients had a newly diagnosed diabetes mellitus at the hospitalization for index event.

Despite regular reinforcement of healthy dietary habits and a significantly higher proportion of patients meeting recommended exercise levels during the first 2 years in the hospital-based follow-up group, we observed no significant difference in the proportion of patients with BMI < 25. Treatment of overweight generally requires more intensive efforts and most studies show that weight regain is common [41].

Compliance to statin therapy was significantly higher in the hospital-based follow-up group during the first 2 years, while to acetylsalisylic acid only at 2-year follow-up. In both groups use of these medications was higher throughout whole study period than described in a study from the Norwegian Myocardial Infarction Register [12]. However, the study still indicates a potential for optimizing secondary preventive medication.

The main strength of this study is the long-term intervention with an individually tailored comprehensive hospital-based follow-up. Selection bias due to socioeconomical status was minimal, given that patient charges in Norway are relatively low. However, this study is limited to one hospital, and not all of the participants had completed the follow-up at the time of data extraction. Generalization of the findings must therefore be done with great caution. The hospital-based follow-up group was younger than the usual care group despite randomization. Consequently, statistical analysis had to be age-adjusted. We assume that the difference might be explained by a higher refusal rate to participate among older persons due to a more demanding follow-up schedule. Smoking status, dietary habits, amount of exercise and use of medications were self-reported, and likely to be affected by reporting bias. As far as the study was designed primarily with regard to the composite endpoint, the results regarding all-cause mortality and secondary outcomes should be interpreted as exploratory and with caution. We assume that open-design of the study implies awareness of participation, which might have influenced the behaviour of participants in both groups. As far as patients in the control group were asked for partipcipation 1 year after the index event to avoid this confounding, only survivors > 12 months were included in data analysis. As a consequence of this limitation, a number of participants included in analysis (n = 1540) was lower than initially planned (n = 1600) to achieve study power of at least 80%. Furthermore, this restricts accessing mortality data during the first year.

The treatment of cardiovascular diseases is improving continuously and there have been amendments in the contemporary practice of managing these patients, particularly as new evidence on the efficacy and safety of antithrombotic therapy is emerging [42]. Cardiovascular event-rates and mortality after MI or coronary intervention are consequently reduced over past decades [27, 28]. Results in a study with long-term follow-up of patients with cardiovascular disease can be therefore influenced as the sample size is based on the mortality and cardiovascular event rates at the time of study start. We assume that new treatment strategies and guidelines are implemented equally in both groups, and would not impact the difference in the outcomes between the study groups per se. However, different follow-up strategies could influence the effect of the prescribed treatment and recommendations given before discharging the patients. Hence, the difference in the achievement of the treatment targets and primary endpoints would expectedly reflect the adherence to the recommendations and quality of the follow-up after the hospitalization for the index event and are the focus of the present study.

## Conclusion

Long-term hospital-based multiple risk factor intervention improved composite endpoint-free survival due to reduced rate of new PCI but did not affect significantly overall mortality. Blood pressure and LDL-cholesterol were most likely to improve through intervention while smoking habits, physical activity and drug adherence to a lesser extent were possible to influence. Frequency of the follow-up consultations seemed to be crucial for maintaining the effect of intervention.

## Supplementary Information

**Additional file 1**. Secondary endpoints in patients with and without hospital-based secondary preventive follow-up program after myocardial infarction, percutaneous coronary intervention or coronary artery bypass grafting.

## Data Availability

The study protocol and data are available on request due to privacy/ethical restrictions, the corresponding author should be contacted in case.

## Abbreviations

BMI: Body mass index
BP: Blood pressure
CABG: Coronary artery bypass grafting
CAD: Coronary artery disease
CVD: Cardiovascular diseases
HbA_1c_: Glycated heamoglobin
LDL: Low-density lipoprotein
MI: Myocardial infarction
PCI: Percutaneous coronary intervention
