# Supplementary material for: Long-term hospital-based secondary prevention of coronary artery disease: a randomized controlled trial
Source: BMC Cardiovasc Disord. 2021 Dec 16;21:600. doi: 10.1186/s12872-021-02426-3 (PMC8679993; doi:10.1186/s12872-021-02426-3)
Supplement: Supplementary file 1 — Additional file 1. Secondary endpoints in patients with and without hospital-based secondary preventive follow-up program after myocardial infarction, percutaneous coronary intervention or coronary artery bypass grafting. [file 12872_2021_2426_MOESM1_ESM.docx]

**Supplemental table**

| **Secondary endpoints in patients with and without hospital-based secondary preventive follow-up program after myocardial infarction, percutaneous coronary intervention or coronary artery bypass grafting.** | | | |
| --- | --- | --- | --- |
|  | **Hospital-based follow-up** | **No hospital-based follow-up** | ***p*** |
| **Proportion of current smokers (%)** |  |  |  |
| Baseline | 28 | 27 | 0.53 |
| 1-year follow-up | 19 | 20 | 0.62 |
| 2-year follow-up | 19 | 20 | 0.65 |
| 5-year follow-up | 14 | 14 | 0.99 |
| 10-year follow-up | 12 | 11 | 0.71 |
| **Mean systolic blood pressure (mmHg)** |  |  |  |
| Baseline | 145 | 147 | 0.1 |
| 1-year follow-up | 132 | 142 | <0.001 |
| 2-year follow-up | 134 | 140 | <0.001 |
| 5-year follow-up | 134 | 139 | <0.001 |
| 10-year follow-up | 136 | 139 | 0.15 |
| **Mean diastolic blood pressure (mmHg)** |  |  |  |
| Baseline | 86 | 87 | 0.5 |
| 1-year follow-up | 81 | 86 | <0.001 |
| 2-year follow-up | 81 | 85 | <0.001 |
| 5-year follow-up | 81 | 83 | <0.001 |
| 10-year follow-up | 82 | 81 | 0.95 |
| **Proportion of patients with blood pressure <140/90 mmHg (%)** |  |  |  |
| Baseline | 36 | 34 | 0.46 |
| 1-year follow-up | 68 | 44 | <0.001 |
| 2-year follow-up | 61 | 47 | <0.001 |
| 5-year follow-up | 62 | 48 | <0.001 |
| 10-year follow-up | 53 | 50 | 0.49 |
| **Mean low-density lipoprotein (LDL) cholesterol (mmol/l)** |  |  |  |
| Baseline | 3.0 | 2.9 | 0.3 |
| 1-year follow-up | 2.1 | 2.3 | <0.001 |
| 2-year follow-up | 2.1 | 2.4 | <0.001 |
| 5-year follow-up | 2.0 | 2.3 | <0.001 |
| 10-year follow-up | 2.1 | 2.1 | 0.99 |
| **Proportion of patients with low-density lipoprotein (LDL) cholesterol <1.8mmol/l (<2.5mmol/l until 1.1.2017) (%)** |  |  |  |
| Baseline | 36 | 36 | 0.9 |
| 1-year follow-up | 79 | 60 | <0.001 |
| 2-year follow-up | 72 | 56 | <0.001 |
| 5-year follow-up | 69 | 46 | <0.001 |
| 10-year follow-up | 43 | 40 | 0.57 |
| **Mean glycated heamoglobin (HbA1c)(mmol/mol) in patients with diabetes mellitus** |  |  |  |
| Baseline | 58 | 57 | 0.56 |
| 1-year follow-up | 57 | 58 | 0.74 |
| 2-year follow-up | 56 | 57 | 0.69 |
| 5-year follow-up | 57 | 55 | 0.34 |
| 10-year follow-up | 57 | 53 | 0.14 |
| **Proportion of diabetes mellitus patients with glycated haemoglobin (HbA1c) <53mmol/mol (%)** |  |  |  |
| Baseline | 45 | 51 | 0.35 |
| 1-year follow-up | 41 | 45 | 0.51 |
| 2-year follow-up | 48 | 50 | 0.81 |
| 5-year follow-up | 46 | 58 | 0.09 |
| 10-year follow-up | 45 | 62 | 0.13 |
| **Mean Body Mass Index (BMI) (kg/m^2^)** |  |  |  |
| Baseline | 28 | 28 | 0.15 |
| 1-year follow-up | 28 | 29 | 0.18 |
| 2-year follow-up | 28 | 28 | 0.53 |
| 5-year follow-up | 28 | 28 | 0.76 |
| 10-year follow-up | 28 | 28 | 0.36 |
| **Proportion of patients with Body Mass Index (BMI) <25 (%)** |  |  |  |
| Baseline | 23 | 24 | 0.59 |
| 1-year follow-up | 27 | 23 | 0.08 |
| 2-year follow-up | 26 | 23 | 0.25 |
| 5-year follow-up | 27 | 25 | 0.36 |
| 10-year follow-up | 28 | 30 | 0.61 |
| **Proportion of patients using statins (%)** |  |  |  |
| 1-year follow-up | 96 | 92 | 0.004 |
| 2-year follow-up | 94 | 90 | 0.002 |
| 5-year follow-up | 91 | 90 | 0.40 |
| 10-year follow-up | 86 | 90 | 0.24 |
| **Proportion of patients using acetylsalicylic acid (%)** |  |  |  |
| 1-year follow-up | 96 | 95 | 0.21 |
| 2-year follow-up | 95 | 92 | 0.04 |
| 5-year follow-up | 87 | 88 | 0.79 |
| 10-year follow-up | 78 | 85 | 0.10 |
| **Proportion of patients reporting >4 days/week of ≥30min physical activity/day (%)** |  |  |  |
| 1-year follow-up | 41 | 30 | <0.001 |
| 2-year follow-up | 38 | 31 | 0.01 |
| 5-year follow-up | 36 | 35 | 0.59 |
| 10-year follow-up | 33 | 26 | 0.22 |
